# Supplementary material for: Effect of statin treatment on metabolites, lipids and prostanoids in patients with Statin Associated Muscle Symptoms (SAMS)
Source: PLoS One. 2023 Dec 15;18(12):e0294498. doi: 10.1371/journal.pone.0294498 (PMC10723679; doi:10.1371/journal.pone.0294498)
Supplement: S3 File — (PDF) [file pone.0294498.s003.pdf]

Supplemental Tables and Figures

S1 Table: Statin Metabolome Cohort Concomitant Meds

S2 Table: Characteristics of Statin Associated Myalgia (SAMS) and Rechallenge Outcome.

S3 Table: Lipid analysis (Positive ionization mode).

S4 Table. Lipid analysis (Negative ionization mode).

S5 Table. Positive ion metabolites identified as differing between cases and controls.

S6 Table. Negative ion metabolites identified as differing between cases and controls

S1 Figure. PCA plot of positive blanks CS and CN

S2 Figure. MSEA analysis Pathways

Supplemental Table S1: Statin Metabolome Cohort Concomitant Meds

Characteristic	CONTROLS (N=39) N (%)	SAMS Cases (N = 28) N (%)	P =
**Antihypertensive/Cardiac Medications**			
Calcium Channel Blockers (Dihydropyridine)	16 (41.0%)	7 (25.9%)	0.206
Calcium Channel Blockers (Non-Dihydropyridine)	2 (5.1%)	0 (0%)	0.232
Alpha Blocker	6 (15.4%)	5 (18.5%)	0.737
Beta Blocker	23 (59.0%)	16 (59.2%)	0.982
Diuretic	8 (20.5%)	5 (18.5%)	0.841
Ace-Inhibitor (ACE-I)	19 (48.7%)	8 (29.6%)	0.121
Angiotensin Receptor Blocker (ARB)	5 (12.8%)	5 (18.5%)	0.526
**Arthritis Medication**			
Nonsteroidal Anti-inflammatory	8 (29.6%)	10 (37.0%)	0.138
**Psychiatric Medications**			
Anxiolytic medication	0 (0%)	2 (7.4%)	0.084
Tricyclic antidepressants	1 (2.6%)	0 (0%)	0.402
Serotonin Re-uptake inhibitors (SSRI)	7 (17.9%)	5 (18.5%)	0.953
Antipsychotic Medication	0 (0%)	2 (7.4%)	0.084
Trazadone	1(2.6%)	0 (0%)	0.402
**Endocrine/Metabolic**			
Vitamin D supplement	19 (48.7%)	20 (74.1%)	**0.039**
Thyroid Medication	4 (10.3%)	3 (11.1%)	0.912
Testosterone replacement	0 (0%)	1 (3.7%)	0.226
Metformin	14 (35.9%)	5 (18.5%)	0.125
Sulfonylurea	2 (5.1%)	0 (0%)	0.232
Glitazone (TZD)	1 (2.6%)	1 (3.7%)	0.791
Insulin	6 (15.4%)	1 (3.7%)	0.130
Fenofibrate	4 (10.2%)	1 (2.6%)	0.064
Colestipol	0(0%)	1 (3.7%)	0.226
Niacin	0 (0%)	1 (3.7%)	0.226
Ezetimibe	3 (7.7%)	8 (29.6%)	**< 0.018**
Praluent (PCSK9 antagonist)	3 (7.7%)	6 (22.2%)	**0.0.09**

Supplemental Table S2: Characteristics of Statin Associated Myalgia (SAMS) and Rechallenge Outcome

Characteristic	CONTROLS (N=39) N (%)	SAMS Cases (N = 28) N (%)	P =
**Statins Associated with Myalgia**			
Rosuvastatin		17 (63.0%)	
Simvastatin		8 (29.6%)	
Fluvastatin		1 (3.7%)	
Atorvastatin		21 (77.8%)	
Lovastatin		4 (14.8%)	
Pravastatin		11 (40.7%)	
Average # statins/patient		2.74 ± 0.17	
**Statin Treatment During Rechallenge Period**			
Atorvastatin	31 (79.6%)	11 (40.7%)	**<0.001**
Rosuvastatin	3 (7.7%)	2 (7.4%)	
Pravastatin	5 (12.8%)	3 (11.0%)	
Simvastatin	1 (2.6%)	2 (7.4%)	
Pitavastatin	0 (0%)	9 (33.3%)	
**Statin Intensity During Rechallenge Period**			
Low	3 (7.7%)	0 (0%)	**<0.001**
Moderate	11 (28.2%)	25 (92.6%)	
High	25 (64.1%)	2 (7.4%)	
Statin Treatment Duration (days)	1326 ± 126	25.74 ± 3.63	**<0.001**
**Statin Myalgia Rechallenge outcomes**			
Myalgia with rechallenge		18 (66.7%)	
Naranjo Score*		8.85 ± 0.95 (Range 7-10)	
NLA SAMS Score*		9.22 ± 2.79 (Range 2-11)	

* Mean +/- Standard Error of Mean

Supplemental Table S3: Lipid analysis (Positive ionization mode).

LipidID	Lipid Name	Lipid Class	Lipid Category	t.stat	p.value	negLOG10(p)	FDR	Direction
posLip76	1_PC(15:0_18:2)+H	PC	Phospholipid	-3.8299	2.98E-04	3.526	0.26634	Increased in CS
posLip1007	4_PC(13:0_20:2)+H | 4_PC(15:0	PC	Phospholipid	-3.7854	3.45E-04	3.4626	0.26634	Increased in CS
posLip1592	4_TG(18:0_20:2_20:5)+NH4 | 4	TG	Glycerolipid	3.694	4.64E-04	3.3336	0.26634	Decreased in CS
posLip72	1_PC(14:0_18:2)+H | 1_PC(16:1	PC	Phospholipid	-3.5789	6.71E-04	3.1736	0.26634	Increased in CS
posLip291	1_TG(17:1_18:1_20:4)+NH4 | 1	TG	Glycerolipid	3.4983	8.65E-04	3.0631	0.26634	Decreased in CS
posLip64	1_LPE(18:2)+H	LPE	Lysophospholipid	-3.4834	9.06E-04	3.0429	0.26634	Increased in CS
posLip660	4_MG(20:1)+NH4 | 4_PEG(n=3_	MG	Glycerolipid	-3.3302	1.45E-03	2.8374	0.27451	Increased in CS
posLip272	1_TG(16:0_20:3_22:5)+NH4 | 1	TG	Glycerolipid	3.3201	1.50E-03	2.8241	0.27451	Decreased in CS
posLip256	1_TG(15:1_16:0_18:1)+NH4 | 1	TG	Glycerolipid	-3.2507	1.85E-03	2.7328	0.27451	Increased in CS
posLip908	4_PA(10:0_26:0)+H | 4_PA(12:0	PC	Phospholipid	-3.2309	1.96E-03	2.707	0.27451	Increased in CS
posLip990	4_PC(13:0_20:3)+H | 4_PC(15:0	PC	Phospholipid	-3.2256	1.99E-03	2.7001	0.27451	Increased in CS
posLip1700	4_TG(20:2_20:2_20:3)+NH4 | 4	TG	Glycerolipid	3.2254	2.00E-03	2.6999	0.27451	Decreased in CS
posLip500	3_PI(36:2)+NH4	PI	Phospholipid	-3.2209	2.02E-03	2.694	0.27451	Increased in CS
posLip310	1_TG(18:1_20:4_22:5)+NH4 | 1	TG	Glycerolipid	3.0997	2.89E-03	2.5384	0.32807	Decreased in CS
posLip885	4_OxTG(14:1(OH)_14:1(OOOO)_	OxTG	Glycerolipid	3.0905	2.97E-03	2.5267	0.32807	Decreased in CS
posLip1490	4_TG(17:1_17:1_18:4)+NH4 | 4	TG	Glycerolipid	3.0739	3.12E-03	2.5056	0.32807	Decreased in CS
posLip357	2_PC(18:2_18:2)+H | 1_PC(18:2	PC	Phospholipid	-3.0107	3.75E-03	2.4263	0.32807	Increased in CS
posLip1086	4_PC(8:0_21:0)+H | 4_PC(10:0_	PC	Phospholipid	-3.0057	3.80E-03	2.4201	0.32807	Increased in CS
posLip1612	4_TG(18:2_18:2_20:2)+NH4 | 4	TG	Glycerolipid	3.0036	3.82E-03	2.4175	0.32807	Decreased in CS
posLip692	4_MGDG(17:2_22:6)+Na	MGDG	Glycerolipid	-2.9833	4.05E-03	2.3922	0.32807	Increased in CS
posLip729	4_OxLPC(22:3(OO))+Na | 4_OxLI	OxLPC	Lysophospholipid	2.979	4.10E-03	2.3868	0.32807	Decreased in CS
posLip1479	4_TG(17:1_17:2_18:4)+NH4 | 4	TG	Glycerolipid	2.9597	4.33E-03	2.3631	0.32807	Decreased in CS
posLip820	4_OxTG(14:0_18:3_24:1(OOO))	OxTG	Glycerolipid	2.9547	4.40E-03	2.3568	0.32807	Decreased in CS
posLip126	1_PE(18:1_20:4)+H | 2_PE(18:1	PE	Phospholipid	2.9152	4.92E-03	2.3083	0.32807	Decreased in CS
posLip1705	4_TG(20:2_20:2_21:0)+NH4 | 4	TG	Glycerolipid	2.8802	5.43E-03	2.2656	0.32807	Decreased in CS
posLip439	2_TG(16:1_16:1_20:5)+NH4 | 2	TG	Glycerolipid	2.8678	5.62E-03	2.2506	0.32807	Decreased in CS
posLip774	4_OxPC(26:0_24:1(Ke))	OxPC	Phospholipid	2.8501	5.90E-03	2.2292	0.32807	Decreased in CS
posLip788	4_OxTG(14:0_16:1_24:1(OOO))	OxTG	Glycerolipid	2.8478	5.94E-03	2.2263	0.32807	Decreased in CS
posLip1085	4_PC(8:0_20:0)+H | 4_PC(10:0_	PC	Phospholipid	-2.8381	6.10E-03	2.2147	0.32807	Increased in CS
posLip1144	4_Plasmanyl-PE(O-20:0/22:5)+H	Plasmalogen	Ether	2.8353	6.15E-03	2.2113	0.32807	Decreased in CS
posLip1712	4_TG(20:2_21:0_22:6)+NH4 | 4	TG	Glycerolipid	2.8333	6.18E-03	2.2089	0.32807	Decreased in CS
posLip799	4_OxTG(14:1(OH)_18:1(OOOO)_	OxTG	Glycerolipid	2.8258	6.31E-03	2.1999	0.32807	Decreased in CS
posLip502	3_PI(38:3)+NH4	PI	Phospholipid	-2.8107	6.58E-03	2.1818	0.32807	Increased in CS
posLip1071	4_PC(20:1_22:6)+H | 4_PC(20:2	PC	Phospholipid	2.8065	6.66E-03	2.1768	0.32807	Decreased in CS
posLip301	1_TG(18:0_20:4_22:4)+NH4	TG	Glycerolipid	2.7956	6.86E-03	2.1637	0.32807	Decreased in CS
posLip567	4_Cer(d18:4_26:0)+H | 4_Cer(d:	Cer	Sphingolipid	2.7867	7.03E-03	2.1532	0.32807	Decreased in CS
posLip350	2_OxLPC(17:2(KeOH))+H	OxLPC	Lysophospholipid	-2.7713	7.33E-03	2.1349	0.32807	Increased in CS
posLip798	4_OxTG(14:0_18:4_24:1(OOO))	OxTG	Glycerolipid	2.7712	7.33E-03	2.1347	0.32807	Decreased in CS
posLip511	4_AcCa(6DC)+H | 4_PEG(n=4_Na	AcCA	Acylcarnitine	2.7475	7.82E-03	2.1067	0.32807	Decreased in CS
posLip1231	4_SM(d15:1_24:1)+H | 4_SM(d1	SM	Sphingolipid	2.7457	7.86E-03	2.1046	0.32807	Decreased in CS
posLip815	4_OxTG(14:1(OH)_14:1(OOOO)_	OxTG	Glycerolipid	-2.7353	8.08E-03	2.0924	0.32807	Increased in CS
posLip580	4_CerG1(d18:1_16:0)+H | 4_Ox	CerG1	Sphingolipid	2.7275	8.26E-03	2.0831	0.32807	Decreased in CS
posLip1039	4_PC(16:1_24:1)+Na | 4_PC(17:	PC	Phospholipid	-2.7273	8.26E-03	2.0829	0.32807	Increased in CS
posLip1416	4_TG(16:0_16:1_18:4)+NH4 | 4	TG	Glycerolipid	2.7154	8.53E-03	2.069	0.32807	Decreased in CS
posLip1157	4_Plasmanyl-PE(O-18:0/22:4)+H	Plasmalogen	Ether	2.7125	8.60E-03	2.0655	0.32807	Decreased in CS
posLip283	1_TG(16:1_18:2_20:4)+NH4 | 1	TG	Glycerolipid	2.6917	9.09E-03	2.0413	0.32807	Decreased in CS
posLip1704	4_TG(20:2_20:2_21:0)+NH4 | 4	TG	Glycerolipid	2.6903	9.13E-03	2.0396	0.32807	Decreased in CS
posLip1586	4_TG(18:0_18:4_18:4)+NH4 | 4	TG	Glycerolipid	2.69	9.14E-03	2.0393	0.32807	Decreased in CS
posLip1080	4_PC(4:1_31:6)+H | 4_PC(5:1_3	PC	Phospholipid	2.6804	9.37E-03	2.0281	0.32807	Decreased in CS
posLip618	4_DG(16:0_22:4)+NH4 | 4_DG(:	DG	Glycerolipid	2.6791	9.41E-03	2.0266	0.32807	Decreased in CS
posLip1398	4_TG(16:0_16:0_18:3)+NH4 | 4	TG	Glycerolipid	-2.676	9.49E-03	2.023	0.32807	Increased in CS

Table shows relative expression of lipids exhibiting a difference between cases and controls at a nominal p value of ≤0.001. Lipids with increased abundance in cases are denoted by blue and decreased lipids by orange.

Supplemental Table S4. Lipid analysis (Negative ionization mode).

Lipid ID	Lipid	Lipid Class	Lipid Category	t.stat	p.value	negLOG10(p)	FDR	Direction
negLip118	2_PC(15:0_18:2)+HCO2 | 1_PC(15:(	PC	Phospholipid	-4.2712	6.69E-05	4.1748	0.028219	Increased in CS
negLip300	4_OxPE(22:0_16:1(COOH))-H | 4_O:	OxPE	Phospholipid	-3.918	2.22E-04	3.6527	0.046939	Increased in CS
negLip120	2_PC(16:0_18:2)+HCO2 | 1_PC(16:(	PC	Phospholipid	-3.6573	5.22E-04	3.2823	0.073437	Increased in CS
negLip416	4_Sulfatide(d14:1_20:0)-H | 4_Sulfa	Sulfatide	Sphingolipid	3.5383	7.62E-04	3.1178	0.080437	Decreased in CS
negLip339	4_OxPE(8:0_13:3(CHO))-H | 4_OxPE	OxPE	Phospholipid	-3.3984	1.18E-03	2.9282	0.083018	Increased in CS
negLip221	4_DMPE(19:0_22:5)-H | 4_DMPE(2(	DMPE	Phospholipid	-3.3982	1.18E-03	2.928	0.083018	Increased in CS
negLip36	1_LPE(18:2)-H	LPE	Lyosphospholipid	-3.3121	1.54E-03	2.8134	0.085244	Increased in CS
negLip131	2_PC(18:0_18:2)+HCO2 | 1_PC(18:(	PC	Phospholipid	-3.2535	1.83E-03	2.7364	0.085244	Increased in CS
negLip40	1_PC(14:0_18:2)+HCO2 | 1_PC(16:1	PC	Phospholipid	-3.2458	1.88E-03	2.7265	0.085244	Increased in CS
negLip140	2_PC(18:2_18:2)+HCO2 | 1_PC(18:2	PC	Phospholipid	-3.2214	2.02E-03	2.6946	0.085244	Increased in CS
negLip296	4_OxPE(22:0_16:1(COOH))-H | 4_O:	OxPE	Phospholipid	-3.1163	2.76E-03	2.5595	0.10032	Increased in CS
negLip75	1_PI(18:0_18:2)-H	PI	Phospholipid	-3.1047	2.85E-03	2.5447	0.10032	Increased in CS
negLip201	4_Cer(d16:1_26:0)+HCO2 | 4_Cer(d	Cer	Sphingolipid	2.9122	4.96E-03	2.3046	0.15224	Decreased in CS
negLip241	4_LPI(20:4)-H	LPI	Lyosphospholipid	2.8938	5.22E-03	2.2822	0.15224	Decreased in CS
negLip253	4_OxPC(20:2_19:4(COOH))+HCO2 |	OxPC	Phospholipid	-2.865	5.66E-03	2.2471	0.15224	Increased in CS
negLip303	4_OxPE(23:0_16:2(COOH))-H | 4_O:	OxPE	Phospholipid	-2.858	5.77E-03	2.2387	0.15224	Increased in CS
negLip73	1_PI(16:0_18:2)-H	PI	Phospholipid	-2.7807	7.14E-03	2.146	0.17735	Increased in CS
negLip418	4_Sulfatide(d15:1_20:0)-H | 4_Sulfatide(d17:0_18:1		Sphingolipid	-2.6771	9.46E-03	2.0243	0.21031	Increased in CS
negLip181	2_Plasmenyl-PE(P-20:0/20:4)-H | 1_	Plasmalogen	Ether	2.6766	9.47E-03	2.0237	0.21031	Decreased in CS

Table shows relative expression of lipids exhibiting a difference between cases and controls at a nominal p value of ≤0.001. Lipids with increased abundance in cases are denoted by blue and decreased lipids by orange.

Supplemental Table S5. Positive ion metabolites identified as differing between cases (CS) and controls.

ID	t.stat	p.value	negLOG10(p)	FDR	Metabolite	m/z	RT (min)	Ion	Direction
posMeta896	-3.8276	0.0003	3.5228	0.11201	2,4-diamino-6-nitrotoluene	168.0767	0.71	M+H	Increased in CS
posMeta319	-3.1378	0.002589	2.5869	0.28992	N-Acetylkynurenine	251.1021	2.68	M+H	Increased in CS
posMeta1593	-2.8375	0.006109	2.214	0.60654	Diaminopimelic acid; Ala-Thr	191.1026	0.92	M+H	Increased in CS
posMeta1339	-2.5901	0.011904	1.9243	0.65444	Glu-Ala	219.0974	1.28	M+H	Increased in CS
posMeta236	-2.4461	0.017247	1.7633	0.65444	L-TYROSINE	182.0809	3.41	M+H	Increased in CS
posMeta725	-2.2805	0.025971	1.5855	0.65444	Pyrroline	70.065	0.78	M+H	Increased in CS
posMeta193	-2.2537	0.027701	1.5575	0.65444	L-TYROSINE	204.0629	3.4	M+Na	Increased in CS
posMeta181	-2.2135	0.030494	1.5158	0.65444	CITRULLINE	159.0762	0.79	M+H	Increased in CS
posMeta54	-2.2099	0.030757	1.5121	0.65444	L-TRYPTOPHAN	243.0533	7.71	M+K	Increased in CS
posMeta582	-2.199	0.031554	1.501	0.65444	Trimethyluric acid	211.0824	7.59	M+H	Increased in CS
posMeta204	-2.165	0.034182	1.4662	0.66015	CITRULLINE	176.1027	0.79	M+H	Increased in CS
posMeta1461	-2.0618	0.043361	1.3629	0.66593	Deoxy-ribitol	159.0631	0.87	M+Na	Increased in CS
posMeta251	-2.0401	0.04554	1.3416	0.66593	L-TRYPTOPHAN	188.0702	7.71	M+H-NH3	Increased in CS
posMeta495	-2.014	0.048283	1.3162	0.66593	Acetylthreonine; aminoadipic acid	162.076	1.12	M+H	Increased in CS
posMeta213	2.0379	0.045762	1.3395	0.66593	L-PROLINE	138.0524	0.86	M+Na	Decreased in CS
posMeta245	2.0452	0.045016	1.3466	0.66593	L-PROLINE	116.0705	0.86	M+H	Decreased in CS
posMeta1048	2.0994	0.039796	1.4002	0.66525	2-trans,4-cis-Decadienoylcarnitine	312.2163	10.67	M+H	Decreased in CS
posMeta7	2.1037	0.039402	1.4045	0.66525	SALICYLAMIDE	121.0283	9.03	M+H	Decreased in CS
posMeta579	2.1186	0.038074	1.4194	0.66015	Salicyluric acid; dopaquinone	196.0603	9.03	M+H	Decreased in CS
posMeta388	2.1244	0.037565	1.4252	0.66015	alpha-Hydroxymetoprolol	284.1851	9.38	M+H	Decreased in CS
posMeta907	2.1381	0.036397	1.4389	0.66015	3-Hydroxybutyrylcarnitine	248.1488	5.75	M+H	Decreased in CS
posMeta206	2.1451	0.035803	1.4461	0.66015	PROPANOYLCARNITINE	218.1384	6.61	M+H	Decreased in CS
posMeta155	2.1618	0.03444	1.4629	0.66015	HEXANOYLCARNITINE	206.1852	9.06	M+H	Decreased in CS
posMeta1010	2.1759	0.033324	1.4772	0.66015	Capryloylglycine; N-acetylaminoactanoic acid	202.1435	10.14	M+H	Decreased in CS
posMeta1829	2.2037	0.031211	1.5057	0.65444	Octenoyl-L-carnitine	286.2007	9.75	M+H	Decreased in CS
posMeta715	2.2273	0.029505	1.5301	0.65444	Ala-Gly	147.0761	8.04	M+H	Decreased in CS
posMeta119	2.2559	0.027557	1.5598	0.65444	4-HYDROXY-L-PROLINE	154.0473	0.75	M+H	Decreased in CS
posMeta36	2.2634	0.027065	1.5676	0.65444	N-ACETYL-L-SERINE	170.0422	1.2	M+H	Decreased in CS
posMeta1742	2.2748	0.026332	1.5795	0.65444	5-aminolevulinic acid; hydroxy-proline	132.0654	1.18	M+H	Decreased in CS
posMeta106	2.2849	0.025698	1.5901	0.65444	CREATININE	152.0217	0.94	M+H	Decreased in CS
posMeta173	2.3302	0.023012	1.638	0.65444	BUTANOYLCARNITINE	232.1539	7.41	M+H	Decreased in CS
posMeta31	2.3461	0.022132	1.655	0.65444	ISOVALERYLCARNITINE	246.1696	8.29	M+H	Decreased in CS
posMeta138	2.3817	0.020266	1.6932	0.65444	L-ASPARAGINE	133.0608	0.71	M+H	Decreased in CS
posMeta178	2.4383	0.017587	1.7548	0.65444	4-HYDROXY-L-PROLINE	132.0654	0.74	M+H	Decreased in CS
posMeta156	2.4683	0.016302	1.7878	0.65444	TAURINE	126.0219	0.73	M+H	Decreased in CS
posMeta292	2.4848	0.01563	1.8061	0.65444	p-cresol glucuronide	307.0781	8.62	M+Na	Decreased in CS
posMeta329	2.6165	0.011107	1.9544	0.65444	2-amino-3-phosphonopropionic acid	170.0212	8.6	M+H	Decreased in CS
posMeta1800	2.6877	0.009192	2.0366	0.65444	Morphine	286.1433	12.69	M+H	Decreased in CS
posMeta101	2.7861	0.00704	2.1524	0.60654	CELLOBIOSE	365.1046	0.8	M+H	Decreased in CS
posMeta139	3.2262	0.001991	2.7009	0.27877	6C-SUGAR ALCOHOL	205.0677	0.74	M+H	Decreased in CS
posMeta1453	3.2361	0.001933	2.7138	0.27877	Ribose; Ribulose; Arabinose	173.042	1.22	M+Na	Decreased in CS
posMeta14	3.2837	0.001675	2.7761	0.27877	GLUTARIC ACID	133.0495	1.2	M+H	Decreased in CS
posMeta30	3.4104	0.001137	2.9443	0.27877	CITRATE	193.0345	1.92	M+H	Decreased in CS

Metabolites in all caps are those that represent level 1 IDs while those in lower case are level 3. Metabolites with increased abundance in cases are denoted by blue and decreased metabolites by orange.

Supplemental Table S6. Negative ion metabolites identified as differing between cases (CS) and controls.

ID	t.stat	p.value	negLOG10(p)	FDR	Metabolite	m/z	RT (min)	Ion	Direction
negMeta123	-2.9453	0.004516	2.3453	0.3119	L-TYROSINE	180.0665	3.4	M-H	Increased in CS
negMeta118	-2.7847	0.007068	2.1507	0.3119	D-GLYCERIC ACID	105.0187	0.82	M-H	Increased in CS
negMeta379	-2.5432	0.013451	1.8712	0.36628	Phosphocreatine	105.0192	1.09	M-H	Increased in CS
negMeta81	-2.5319	0.013852	1.8585	0.36628	CITRULLINE	210.0666	0.79	M+Cl	Increased in CS
negMeta120	-2.4712	0.016183	1.7909	0.37441	CITRULLINE	174.0883	0.79	M-H	Increased in CS
negMeta1143	-2.4364	0.017675	1.7526	0.37441	Ornithine	131.0826	0.79	M-H	Increased in CS
negMeta562	-2.3876	0.019968	1.6997	0.37441	LYSOPE 18:2	476.2781	13.04	M-H	Increased in CS
negMeta1183	-2.3315	0.022937	1.6395	0.38744	Acetylglycine; Aspartate-semialdehyde	116.0351	1.81	M-H	Increased in CS
negMeta6	-2.1189	0.038044	1.4197	0.47439	HOMOVANILLATE	181.0526	8.41	M-H	Increased in CS
negMeta36	-2.1044	0.039336	1.4052	0.47439	SUCCINATE	257.0282	2.25	M-H	Increased in CS
negMeta1120	-2.0737	0.042203	1.3747	0.47439	Indole-3-carboxyaldehyde	144.0454	9.82	2M+Na-2H	Increased in CS
negMeta51	-2.0542	0.044108	1.3555	0.47439	L-ARGININE	209.0812	0.73	M-H	Increased in CS
negMeta115	-2.0525	0.044276	1.3538	0.47439	SUCCINATE	117.0191	2.32	M-H	Increased in CS
negMeta113	-2.0224	0.047389	1.3243	0.49591	sedoheptulose; trimethyluric acid	209.0666	0.84	M-H	Increased in CS
negMeta1161	1.9994	0.049877	1.3021	0.49591	Phenyllactic acid; homovanillin; 2-hydroxy-2-phenylpropanoic acid	165.0555	8.98	M-H	Decreased in CS
negMeta95	2.0527	0.044260	1.354	0.47439	3-HYDROXYBENZOATE	137.0244	10.04	M-H	Decreased in CS
negMeta27	2.1289	0.037176	1.4297	0.47439	3-HYDROXY-3-METHYLGLUTARATE	161.0455	3.03	M-H	Decreased in CS
negMeta910	2.1478	0.035582	1.4488	0.47439	3-oxododecanoic acid	213.1494	12.09	M-H	Decreased in CS
negMeta86	2.1642	0.034246	1.4654	0.47439	D-GLUCONATE	177.0403	0.74	M-H	Decreased in CS
negMeta46	2.204	0.031189	1.506	0.46795	DIHYDROXYMANDELIC ACID	205.0116	10.04	M-H	Decreased in CS
negMeta94	2.3749	0.020608	1.686	0.37698	L-CARNITINE	196.0742	0.97	M+Cl	Decreased in CS
negMeta69	2.3902	0.019843	1.7024	0.37441	2-HYDROXYPHENYLACETIC ACID	151.0399	8.62	M-H	Decreased in CS
negMeta175	2.3952	0.019597	1.7078	0.37441	alpha-ketooctanoic acid	157.0867	9.13	M-H	Decreased in CS
negMeta205	2.4033	0.019206	1.7166	0.37441	Galloylglycerol	243.0491	7.76	M-H	Decreased in CS
negMeta1200	2.4313	0.017899	1.7472	0.37441	Hydroxyoctanoic acid; hydroxycaprylic acid	159.1025	9.77	M-H	Decreased in CS
negMeta744	2.4577	0.016745	1.7761	0.37441	5-hydroxyvaleric acid; 5-hydroxypetanoic acid	117.0557	4.94	M-H	Decreased in CS
negMeta87	2.4592	0.016682	1.7777	0.37441	3-Hydroxydecanoic acid	187.1338	11.36	M-H	Decreased in CS
negMeta1179	2.6807	0.009365	2.0285	0.31928	5-hydroxyvaleric acid; 5-hydroxypetanoic acid	117.0556	4.12	M-H	Decreased in CS
negMeta730	2.696	0.008991	2.0462	0.31928	Tiglic acid; senecioic acid; 3-methylbut-3-enoic acid	99.0451	7.36	M-H	Decreased in CS
negMeta1054	2.7118	0.008615	2.0648	0.31928	p-cresol glucuronide; 2-benzoyl-glucose	283.0819	8.59	M-H	Decreased in CS
negMeta71	2.7845	0.007070	2.1506	0.3119	2-HYDROXYPHENYLACETIC ACID	151.0398	8.68	M-H	Decreased in CS
negMeta883	2.865	0.005660	2.2472	0.3119	N-acetyl-S-(N-methylcarbamoyl)cysteine	219.045	0.75	M-H	Decreased in CS
negMeta63	2.8656	0.005650	2.2479	0.3119	ASCORBATE	193.0351	0.87	M-H	Decreased in CS
negMeta916	2.888	0.005307	2.2751	0.3119	5-aminolevulinic acid; 3-hydroxy-proline; acetyl-alanine; propionylglycine	130.0507	1.06	M-H	Decreased in CS
negMeta61	3.0223	0.003624	2.4409	0.3119	CELLOBIOSE	377.0853	0.81	M+Cl	Decreased in CS
negMeta49	3.7057	0.000447	3.3501	0.16747	2-HYDROXYPHENYLACETIC ACID	151.0399	8.51	M-H	Decreased in CS
negMeta1	3.9922	0.000174	3.7605	0.13017	PYRUVATE	221.0301	1.37	2M+FA-H	Decreased in CS

Metabolites with increased abundance in cases are denoted by blue and decreased metabolites by orange

Supplemental Figure S1. PCA plot of positive blanks CS and CN

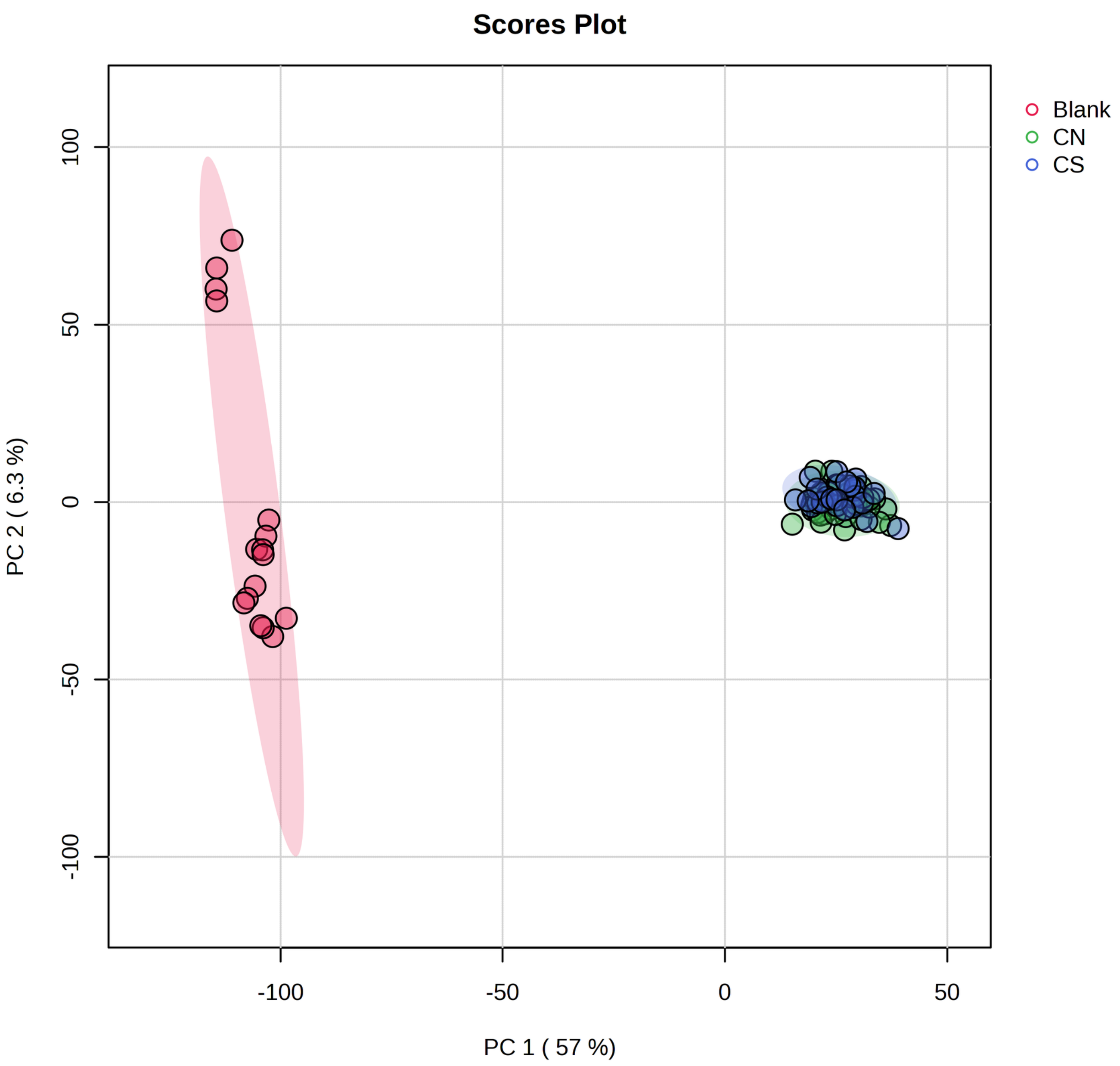

Supplemental Figure S2. MSEA analysis Pathways

A. MSEA Analysis Pathways Upregulated in Cases

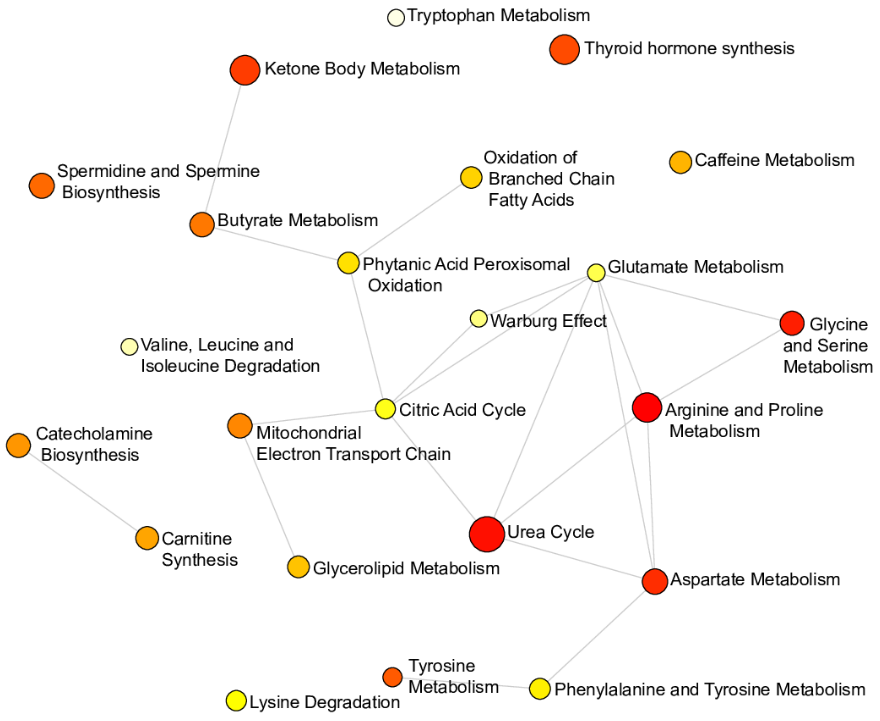

B. MSEA Analysis Pathways Downregulated in Cases

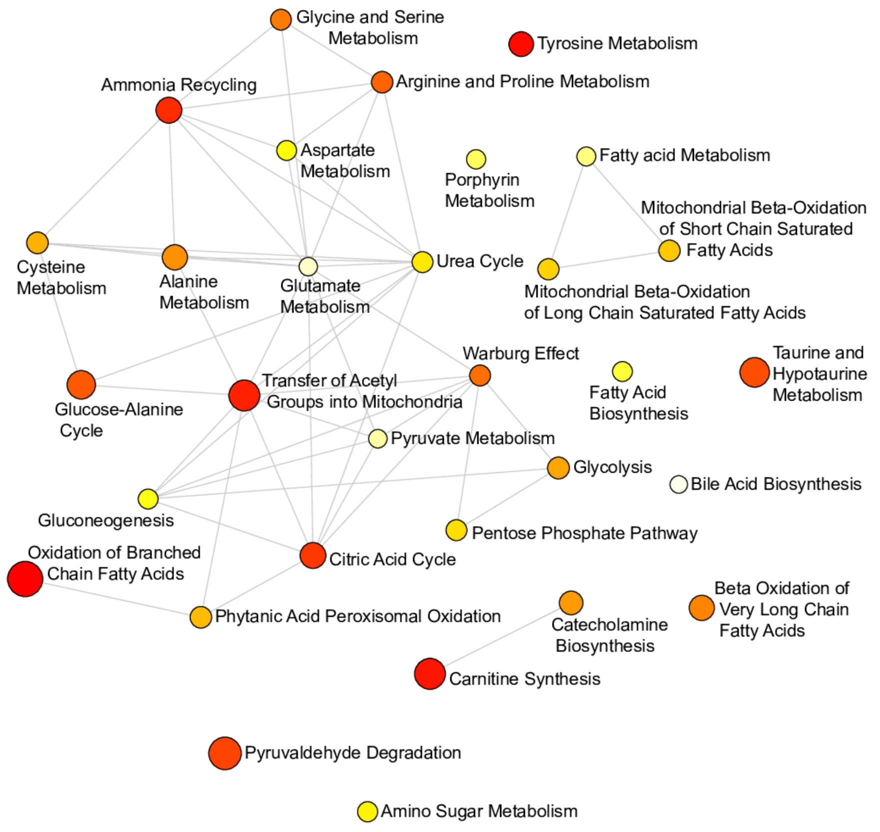

*see figure 2 in manuscript for tabular information)
