# Supplementary material for: Effect of statin treatment on metabolites, lipids and prostanoids in patients with Statin Associated Muscle Symptoms (SAMS)
Source: PLoS One. 2023 Dec 15;18(12):e0294498. doi: 10.1371/journal.pone.0294498 (PMC10723679; doi:10.1371/journal.pone.0294498)
Supplement: Attachment — Submitted filename: Comments for statin paper.docx [file pone.0294498.s004.docx]

The manuscript titled ‘**Effect of Statin Treatment on Metabolites, Lipids and Prostanoids in Patients with Statin Associated Muscle Symptoms (SAMS)**” explored plasma metabolomics and lipidomics to understand pathophysiology of SAMS, an adverse effect of statin therapy, and to identify biomarkers for better diagnosis. The results provided insights into the pathogenesis of myalgia and muscle weakness experienced by patients on re-exposure to statin therapy. The authors suggested that alterations in pro-inflammatory lipids and impaired mitochondrial related energy metabolism underlie the muscle symptoms of patients with statin associated muscle symptoms (SAMS) as reflected in change in LCMS intensities. However, there are still some concerns.

1) The figures provided are very low resolution with no visibility of annotated texts, hence it makes difficult for the reviewer to review the manuscript carefully.
2) It is suggested to follow the same format throughout the text, for example, acylcarnitines is mentioned as ‘acyl-carnitines’ at some places. Similarly, TCA cycle is mentioned as Krebs cycle at many places.
3) Page 21, Line 441 “SAMS patients had decreases in ceramides….” should be “SAMS patients had decrease in ceramides….”
4) Page 23, Line 490, two commas after succinate.
5) The authors are requested to follow uniform pattern for titles. For example, the subtitle “Study population” under main title “MATERIALS AND METHODS” is bold, not italic, and smaller font size while under the main title “RESULTS” is bold and italics.
6) How does authors explain the effect of diet/microbiome on lipids and metabolites as they may induces significant differences in the profiles.
7) Did the authors find gender-based differences in lipid/metabolic profile.
8) Although the introduction states that the study is intended to explore the biomarkers for the disease, the authors have not mentioned any lipid/lipids/metabolites which can be used as biomarkers conclusively for disease diagnosis.
9) It is suggested to use any PCA/PLS-DA/OPLS-DA modelling to show whether the two groups can be differentiated based on the features identified by LCMS profiling and use ROC analysis to comment on diagnostic accuracy of significant lipid/metabolite as a biomarker for the disease.
