# Supplementary material for: Effect of statin treatment on metabolites, lipids and prostanoids in patients with Statin Associated Muscle Symptoms (SAMS)
Source: PLoS One. 2023 Dec 15;18(12):e0294498. doi: 10.1371/journal.pone.0294498 (PMC10723679; doi:10.1371/journal.pone.0294498)
Supplement: Attachment — Submitted filename: Response to Reviewers FINAL.doc [file pone.0294498.s005.doc]

**College of Medicine**

Department of Pharmacology

Room 215 TSRB Building

71 S. Manassas Street

Memphis, TN 38103

Tel: (901) 448-6000

Fax: (901) 448-7206

THE UNIVERSITY OF TENNESSEE

**Health Science Center**

February 20, 2023

Anil Bhatia, Ph.D.

Academic Editor

PLOS ONE

Dear Dr. Bhatia,

Thank you for the invitation to revise and resubmit our manuscript “Effect of Statin Treatment on Metabolites, Lipids and Prostanoids in Patients with Statin Associated Muscle Symptoms (SAMS). We also thank you and the reviewers for your helpful and insightful comments. We are gratified that you appreciate that this investigation is novel and provides important insights into this common clinical entity. In this “Response to Reviewers” we respond to each point raised by yourself and the reviewers as outlined below.

Academic Editor Comments:

*1. “funding information should not appear in the Acknowledgements section or other areas of your manuscript”*

**Response: We have removed references to funding mechanism from the manuscript. The funding statement is correct as written.**

*2. “Statement of competing interests”*

**Response: The authors have declared that no competing interests exist.**

*3. “Data Availability”*

**Thank you for updating our Data Availability statement to read: “Supporting information files will be uploaded to metabolomics workbench (**[**https://www.metabolomicsworkbench.org/**](https://www.metabolomicsworkbench.org/)**). Batch files for MZmine processing are included in the manuscript as supplemental information.**

*4. “Proofreading is needed”*

**We have carefully proofread the manuscript and corrected any errors, grammatical or otherwise.**

*5. “I suggest adding a data acquisition/sample injection sequence file in the supplementary”*

**We thank the reviewer for this comment. We don’t typically include the sequence files as supplemental information as it can be very long. In the case for this data, we analyzed each data set as 4 batches. Since each data was analyzed in both positive and negative ion modes as well as both lipids and metabolites, we would have 16 different sequence lists. All of our runs begin with 3 blank injections followed by a neat QC that consists of amino acids, a red cross plasma QC and an extraction blank. These are then followed by 10 randomized patient sample blocks. In between each block of 10 samples, we analyze a blank, a neat QA, a red cross plasma and an extraction blank.**

*6. “On line 185, metabolomics internal standards solution. . . Specify with a catalog number”*

**We thank the reviewers for this comment. The internal standard solution is a mixture that we prepare following our previous work. We have added the appropriate reference. (**[**https://journals.plos.org/plosone/article?id=10.1371/journal.pone.0222393**](https://journals.plos.org/plosone/article?id=10.1371/journal.pone.0222393) **)**

*7. “How was the data normalized before the feature identification, TIC or internal standard”*

**We added a detailed data preprocessing description to the ‘Data preprocessing” section and includes that we used probabilistic quotient normalization.**

*8. “Has the applied 30% CV cutoff in pool QC?”*

**We are not sure what this refers to. We will be happy to respond if you can clarify this.**

*9. “necessary information about post-data treatment is missing”*

**We are not sure what this refers to. We will be happy to respond if you can clarify this.**

*10. “provide the parameters used in mZmine in supplementary”*

**We have included the MZmine batch files for metabolomic processing as supplemental information. The Lipidomics processing used the default setting of LipidMatch.**

**Reviewer #1**

*1. “details on the blood sample collection were not provided . . . have the authors collected the fasting blood samples to avoid diet induced changes?”*

**Blood samples were collected following an overnight fast (8-12 hours). This information has been added to the Materials and Methods section. Thank you for bringing this to our attention.**

2. “*did the author perform the PCA plots as a part of multivariate data analysis for the metabolomic and lipidomic data?”*

**Yes, we performed PCA analysis initially to evaluate clustering. We did not include the PCA plots as we primarily focused our analyses on heatmap clustering and t-test analysis along with the updated ROC work.**

*3. “What are the units for the Y-axis values presented in the Figure 1A and 1B? please provide P-values for each negative and positive ion”*

**The units for the box plots are arbitrary as these are normalized values based on our data preprocessing procedure. The P-values are presented in supplementary tables 3 and 4.**

*4. “Have the authors performed validation studies to confirm the identity of the identified metabolites and lipids with authentic standards?”*

**For metabolites, we provided the identification confidence based on the capitalization of the metabolite. Metabolites that are in ALL CAPS have been identified with reference to an authentic standard while all others are from a database search.**

**For lipids, we identify lipids based on MS/MS confidence. A ‘1’ preceding the lipid name indicates that the lipid was identified with sufficient MS/MS data to identify the fatty acyl chains, a value of 2 just indicates that we used all ion fragmentation MS/MS data to identify the fatty acyl chains. A value of 3 means that we only identified the head group and a value of 4 means that we identified based on a reference to a lipid database.**

**We add text to the metabolomic and lipidomic sections to clarify this.**

*5. “It is important to perform the validation of the identified metabolites by Receiver Operating Characteristic (ROC) analysis for evaluating the accuracy of a statistical model.”*

**We thank the reviewers for this suggestion. We have now conducted ROC analysis and include 2 additional figures (figs 3 and 4) with the results from this analysis. In addition, we have added the details of our analysis to the methods section in the “data preprocessing section”. We have also added a paragraph in the results section entitles “ROC curve biomarker analysis”. Finally, we expanded the discussion to include a section on “Biomarker Analysis”.**

*6. “All the figures presented in the manuscript are poor and needs high resolution figures”*

**We provide PNG files derived from the original figures. These should be suitable for review and publication.**

**Reviewer #2.**

*1. “It is recommended to use modeling features and more rigorous analysis to comment on the diagnostic accuracy of the biomarkers.”*

**Thank you for this recommendation. We have added the ROC analysis as outlined in our response to comment 5 by reviewer one above.**

*2. “The authors have not satisfactorily explained the exact mechanism/reason for the increase/decrease seen in lipids or metabolites.”*

**We thank the reviewer for bringing this to our attention. Although the exact triggering mechanism for the observed changes is difficult to discern, the weight of evidence generated by this metabolomic analysis points to a central role of disordered mitochondrial energy metabolism in the genesis of statin associated muscle symptoms. We have expanded the Conclusions paragraph to more fully explore the insights into the mechanism(s) underlying the observed changes and have added a figure (Figure 8) that summarizes the changes in mitochondrial metabolism.**

**Additional comments:**

***3. “****The figures provided are very low resolution with no visibility of annotated texts”*

**This issue was also raised by reviewer one. Please see our response to reviewer one comment #6 above**

*4. “It is suggested to follow the same format throughout the text, for example acycarnitines is mentioned as ‘acyl-carnitines in some places. Similarly the TCA cycle is mentioned as Krebs cycle at many places”*

**The manuscript now uses uniform format for both acylcarnitines and TCA cycle.**

*5. Page 21, Line 441 “SAMS patients had decreases in ceramides….” should be “SAMS patients had decrease in ceramides….”*

**The suggested changes have been made**

*6. Page 23, Line 490, two commas after succinate.*

**The suggested changes have been made**

*7. The authors are requested to follow uniform pattern for titles. For example, the subtitle “Study population” under main title “MATERIALS AND METHODS” is bold, not italic, and smaller font size while under the main title “RESULTS” is bold and italics*

**The suggested changes have been made.**

*8. “How does authors explain the effect of diet/microbiome on lipids and metabolites as they may induce significant differences in the the profiles?”*

**The study participants were not placed on a special diet prior to obtaining blood samples. As lipid clinic patients they received uniform dietary recommendations regardless of which group they were in (statin tolerant vs intolerant). Although we cannot exclude differences in dietary intake between individual participants, we do believe that on average, dietary profiles were comparable in both groups. We have added this discussion to the “limitations” paragraph.**

*9. “Did the authors find gender-based differences in lipid/metabolic profile?”*

**We did not perform gender-based analysis given the small number of women participants. We only had two controls that were female so it would be difficult to conduct the analysis.**

*10. “Although the introduction states that the study is intended to explore the biomarkers for the disease, the authors have not mentioned any lipid/lipids/metabolites which can be used as biomarkers conclusively for disease diagnosis.”*

**Thank you for this helpful comment. As we have described in our conclusion, we have reported changes in including carnitine, acylcarnitines, caproyl glycine, pyruvate, ribose, and coenzyme A. Also Please see response to Reviewer 1 comment #5 above.**

*“It is suggested to use any PCA/PLS-DA/OPLS-DA modelling to show whether the two groups can be differentiated based on the features identified by LCMS profiling and use ROC analysis to comment on diagnostic accuracy of significant lipid/metabolite as a biomarker for the disease?”*

**We conducted ROC curve analysis using support vector machine learning (SVM). Details of this have now been included in the manuscript as described in our response to Reviewer 2 comment #1 above.**
