# Supplementary material for: Effect of statin treatment on metabolites, lipids and prostanoids in patients with Statin Associated Muscle Symptoms (SAMS)
Source: PLoS One. 2023 Dec 15;18(12):e0294498. doi: 10.1371/journal.pone.0294498 (PMC10723679; doi:10.1371/journal.pone.0294498)
Supplement: Attachment — Submitted filename: Comments for Plosone.docx [file pone.0294498.s006.docx]

The authors have done great job in the manuscript. Experimental design is very clever. Classification and consideration of patients is impressive. Authors have selected individuals very carefully by considering parameters to support the study. They have omitted the patient with important conditions that may hinder their observations.

Authors have done great job in mentioned very basic metabolites and pathways. Their interpretations are awesome. Authors have done great job in mentioning about the strengths and limitations of the study.

Overall impression of an article is very impressive.

There are some revisions that they can focus on to make the manuscript readable :

1. Table 3 second line, 4^th^ column there is a spelling mistake. Carefully, please fix it.
2. Please, make the figures 1A, 1B, 2A and 2B clearer so that reading is easier to read.
3. Figure 4 and Figure 5 are very hard to read. If authors can improve on the clarity of the Figures, reader will enjoy and understand the pathways better.
