# Supplementary material for: Effect of statin treatment on metabolites, lipids and prostanoids in patients with Statin Associated Muscle Symptoms (SAMS)
Source: PLoS One. 2023 Dec 15;18(12):e0294498. doi: 10.1371/journal.pone.0294498 (PMC10723679; doi:10.1371/journal.pone.0294498)
Supplement: Attachment — Submitted filename: review comments.docx [file pone.0294498.s007.docx]

**Effect of Statin Treatment on Metabolites, Lipids and Prostanoids in Patients with Statin Associated Muscle Symptoms (SAMS)**

Reviews

1. The discussion about the mitochondrial metabolites has to be elaborated more and to be accompanied by recent references

1.Page 30, Line 660... …. “Instead of rigorous selection….” Should be “the rigorous selection” likewise line 676 … “the functional”. The author are requested to check the grammatical errors.Justification is also required.

2. In line 680 consider changing the wording “prior to” …. “Before”

3. In line 666…. statin associated should be accompanied by hyphen as “statin-associated.”

4. “The figures are not clear and requires more visibility”.
